# Contribution of Word Reading Speed to Reading Comprehension in Brazilian Children: Does Speed Matter to the Comprehension Model?

**DOI:** 10.3389/fpsyg.2017.00630

**Published:** 2017-04-20

**Authors:** Alessandra G. Seabra, Natália M. Dias, Tatiana Mecca, Elizeu C. Macedo

**Affiliations:** ^1^Developmental Disorders Program, Universidade Presbiteriana MackenzieSão Paulo, Brazil; ^2^Educational Psychology Post-Graduation Program, Centro Universitário FIEOOsasco, Brazil

**Keywords:** reading competence, fluency, learning, cognitive assessment, cognitive models

## Abstract

Studies have suggested that reading speed (RS) or fluency should be a component of reading comprehension (RC) models. There is also evidence of a relationship between RS and RC. However, some questions remain to be explored, as the changes in such a relationship may be a function of development. In addition, while there are studies published with English speakers and learners, less evidence exists in more transparent orthographies, such as Portuguese. This study investigated the relationship between RC and RS in typical readers. Objectives included elucidating the following: (1) the contribution of RS to RC controlling for intelligence, word recognition, and listening and (2) the differential relationships and contributions of RS to comprehension in different school grades. The sample of participants comprised 212 students (*M* = 8.76; *SD* = 1.06) from 2nd to 4th grade. We assessed intelligence, word recognition, word RS, listening, and RC. Performance in all tests increased as a function of grade. There were significant connections between RC and all other measures. Nonetheless, the regression analysis revealed that word RS has a unique contribution to RC after controlling for intelligence, word recognition, and listening, with a very modest but significant improvement in the explanatory power of the model. We found a significant relationship between RS and RC only for 4th grade and such relationship becomes marginal after controlling for word recognition. The findings suggest that RS could contribute to RC in Portuguese beyond the variance shared with listening and, mainly, word recognition, but such a contribution was very small. The data also reveal a differential relationship between RS and RC in different school grades; specifically, only for the 4th grade does RS begins to relate to RC. The findings add a developmental perspective to the study of reading models.

## Introduction

The National Reading Panel highlights three important areas for reading, learning and competence: alphabetics, which is related to word recognition skills; fluency, the ability to read with speed, accuracy, and proper expression (prosody); and comprehension, here understood as reading comprehension (RC) or reading competence, a complex process that integrates other abilities such vocabulary comprehension and strategies for understanding. In this statement by the National Reading Panel, it is clear that the notion of independent yet correlated components exists and recognizes that fluency is related to word recognition skills, but its position is clear about the independence of such processes — that is, word recognition does not necessarily lead to fluency (National Reading Panel (US) and National Institute of Child Health and Human Development (US), 2000). A relatively recent review on the theme-compiled studies in the area has corroborated the view of fluency as a concept that integrates accuracy, automaticity, and prosody in oral reading (Kuhn et al., 2010).

This componential view of reading is also represented in cognitive models such as the Simple View of Reading (SVR), a model proposed by Gough and Tunmer (1986) that suggests that reading competence (R) can be explained by two components, decoding (D) and listening comprehension (LC), which is expressed by the formula R = D × LC. A modified and elaborated version is the component model of reading (CMR; Aaron et al., 2008). CMR postulates that three domains can impact reading learning and competence: (1) the cognitive domain, comprising word recognition and comprehension (similar to SVR, but note that the “word recognition” component expands the idea of decoding); (2) the psychological domain, which includes motivation, interest, learning styles and other psychological phenomena; and (3) the ecological domain, including variables such as culture, home and classroom environments.

Considering the cognitive components (SVR or cognitive domain of CMR), evidence already suggests that decoding and LC can explain RC in different orthographies. For example, with students from 2nd, 3rd, and 4th grades, approximately 60% of the variance in RC was explained by decoding and listening for Spanish speakers, while approximately 50% of the variance in RC was explained for English speakers. Spanish is considered as having a transparent orthography due to the close relationship between phonemes and graphemes. On the other hand, English has an opaque orthography, in which there is a lack of one-to-one correspondence between sound and the letter. Another interesting finding shows that the contribution of components to RC in Spanish 3rd graders and English 4th graders was very similar. Authors interpreted this result as Spanish-speaking children mastering decoding skills earlier than English-speaking children, who use a more opaque orthography. The same study investigated the model in Chinese orthography, which has morphosyllabic orthography (in which a character represents a word). Some similar patterns were found, with component skills accounting for 31% of the variance in Chinese RC at 2nd grade and 42% at 4th grade. Despite this, the explanatory power of the model for Chinese was smaller than in English and in Spanish (Joshi et al., 2012).

Expanding this view and considering the suggestion of an additional component to the SVR model by Joshi and Aaron (2000), speed has also been studied as a new and independent skill that contributes to reading competence. Indeed, in the original work of Joshi and Aaron (2000) with 3rd graders, D and LC accounted for 48% of variance in RC, and the addition of the speed component, which was assessed in a letter-naming task, added 10% to the explanatory power of the model, suggesting some unique contribution of the speed component. More recently, Aaron et al. (2008) supported word recognition and linguistic comprehension as the main components of cognitive domains of reading (explaining 37 to 41% of RC from 2nd to 5th grade), while speed (that authors referred as processing speed, assessed by a letter-naming task) shows some inconsistent contributions that can vary from 11% in 2nd grade to 2.5% in 5th grade. Such findings suggest that there is a decreasing trend in the effect of speed on RC with development. Another study with 4th and 5th graders also found some significant and unique contribution from speed to RC, as assessed in a picture-naming task, but such a contribution was small, varying from 2% for 4th grade and 1.4% for 5th grade. Authors argue that such a low contribution is due to the speed that has already had an effect upon decoding (Johnston and Kirby, 2006).

Other studies had investigated the same question — that is, the cognitive components or contributors of competent reading — but used a different measurement than speed, which was reading fluency itself. According to Kuhn et al. (2010), reading fluency combines accuracy, automaticity, and prosody. In fact, studies have investigated one or more of these aspects of fluency in different units of reading, such as words or texts, with some contradictory results.

With a sample of 5th grade students from 11 to 12 years of age, Turkyılmaz et al. (2014) found that, despite all fluency measures that were significantly related with RC, oral reading fluency had the strongest contribution to prediction of RC when compared to silent reading fluency and retell fluency. Similarly, Klauda and Guthrie (2008) investigated the relationships between three measures of fluency (at the word, syntactic – phrase and sentence units of text, and passage levels) and RC in 5th grade students. The authors found that three types of fluency were individually related to performance on a RC test. In addition, some evidence suggested that RC and reading fluency at the syntactic level appeared to have a bidirectional relationship. The authors suggest that fluency and comprehension becomes more similar over time up to the age of approximately 10 or 12 years. In this sense, they argue that it may be useful to examine the existence of different relationships between fluency (and different fluency skills) and comprehension across different grade levels. Furthermore, a national (Brazilian) study that evaluates prosody found no significant relationship with student RC from 3rd to 5th grades and only marginal non-significant trends in this relationship for 3rd and 4th grade students (Martins and Capellini, 2014).

Despite the relevance of fluency for reading competence (National Reading Panel (US) and National Institute of Child Health and Human Development (US), 2000; Hudson et al., 2005), studies in the area show that there is less agreement as to how to assess such skills, as some studies assessed naming speed (Joshi and Aaron, 2000; Johnston and Kirby, 2006; Aaron et al., 2008), some assessed word or different units of text reading rates (Klauda and Guthrie, 2008; Turkyılmaz et al., 2014), and others assess prosody (Martins and Capellini, 2014). This fact can lead to difficulty in making comparisons between the findings in the area because there is no clarity regarding the independence or overlap between these several measurements. Additionally, aspects of reading fluency had been poorly studied in Brazil (Puliezi and Maluf, 2014). In this context, we evaluated accuracy and speed at the word level [that is, the reading of isolated words and word reading speed (RS), respectively], listening and RC at the sentence level. We choose these particular levels of analyses because the use of more complex levels could imply more demands on the tasks, such as working memory on comprehension of longer text, and also because we intend to investigate the relationship between such skills in children in the first years of elementary school, in which competence at the text level could not be consolidated.

Previous findings in Brazil elucidated the development of listening and RC of sentences, as well as word reading strategies. Dias et al. (2015) found that both listening and RC developed in 1st to 3rd grade, with no difference between 3rd and 4th grades in the tests. Regarding word reading strategies, the study suggested further development of alphabetical (decoding or phonological route) and logographic (contextual word recognition) strategies in early literacy (mainly in 1st and 2nd grades), and further development of orthographic processing in more advanced grades (3rd and mainly 4th grades). In this sense, one wonders if, with the proficiency in word recognition and development of orthographic reading, RS grows. Therefore, it can be expected that faster reading with educational progression is associated with the automation of alphabetic reading and use of orthographic reading and, consequently, with better RC. In this case, while in the early years of elementary school, word recognition would be the most important skill for RC, and with educational progression, fluency would become more relevant.

Therefore, more studies are needed and some questions remained to be further explored. For example, while there are studies that examined English middle-school student speakers/learners, less evidence exists in other orthographies, such as Portuguese, and studies of earlier grades. In this context, this study investigated the relationship between sentence RC and word RS in typical readers during elementary school. Specifically, we wished to elucidate (1) the contribution of RS to RC after controlling for intelligence, word recognition, and listening and (2) the differential relationships and contributions of RS to comprehension in different school grade levels. Our hypotheses are (1) word RS will present a modest but significant contribution to the explanatory model of RC, showing some unique contributions not accounted for by intelligence, word recognition, and listening and (2) more consistent relationships and contributions will be established between RS and comprehension in the earlier grades in our sample.

## Materials and Methods

### Participants

The participants comprised 223 students from 2nd to 4th grade in São Paulo, Brazil. From this initial sample, 11 participants were excluded (10 with histories of academic failure and 1 with an indicator of intellectual disability as assessed by the Raven test). The final sample comprised 212 students with 51.4% female (Mean age = 8.76 years; *SD* = 1.06), and included 85 students from the 2nd grade (Mean age = 7.92; *SD* = 0.727); 52 students from the 3rd grade (Mean age = 8.65; *SD* = 0.623), and 75 students from the 4th grade (Mean age = 9.80; *SD* = 0.658). In the final sample, there were no students with motor or sensorial disabilities that would impair their performance in the tests.

### Instruments

#### Words and Non-words Reading Competence Test – WNw-RCT

The WNw-RCT (Seabra and Capovilla, 2010) assesses competence in reading isolated words and is comprised of 70 test items, each of which features a picture paired with a written word. There are seven different types of items: correct regular words [CR, e.g., the word ‘FADA’ (fairy in English) with the image of a fairy], correct irregular words [CI, e.g., the word ‘BRUXA’ (witch) with the image of a witch]; semantic changes [SC, e.g., the word ‘RÁDIO’ (radio in English) with the image of a phone]; visual changes [VC, e.g., the word ‘TEIEUISÃO’ (the correct spelling is TELEVISÃO) with the image of a television]; phonological changes [PC, e.g., the word ‘MÁCHICO’ (the correct spelling is MÁGICO) with the image of a wizard]; weird non-words [WN, e.g., the word ‘MELOCE’ (a word that does not exist in Portuguese)], and homophone non-words [HN, e.g., the word ‘TACSI’ (the correct spelling is TAXI) with the image of a taxi]. The children need to choose the corrected word and reject any semantic errors or non-words. Despite allowing for the differential assessment of reading strategies (logographic, alphabetic, and orthographic), we used the total score in this study, an index of word-recognition skills.

#### Contrastive Test of Listening and Reading Comprehension – CTLRC

The CTLRC (Capovilla and Seabra, 2013) assesses listening and RC skills. The instrument consists of two subtests: RC and LC, each with 40 test items arranged in order of increasing difficulty. For each item, the child must choose between five alternative figures, the one that corresponds to the sentence heard in the case of the LC subtest or read in the case of the RC.

#### Reading Speed Test – RST

Reading Speed Test (Montiel, 2008) was used to assess RS. The test requires the subject to read isolated words presented in the middle of the computer screen as quickly as possible. Test scores show the successes and mistakes and the time required for reading (speed measure). The RST consists of 60 items divided into four parts (P1 to P4): 15 irregular words (P1), 15 pseudo-words (P2), 15 words related to content (i.e., nouns _ P3) and 15 words related to function (such as conjunctions, adjectives, and adverbs _ P4). In this study, we used only parts 1 and 2 (thus, 30 items). All of the words containing three to four letters were presented using the Times New Roman font, size 72 in black ink, and for an indefinite time on the screen. Only the times for items resulting in 100% reading accuracy were considered in the analysis.

#### Raven’s Colored Standard Progressive Matrices – RCSPM

The RCSPM assess general intellectual ability (Angelini et al., 1999), specifically, the reasoning related to the formation of new creative insights and high-level functions.

### Procedure

Our study was approved by the Research Ethics Committee. Agreement Terms were sent to the students’ parents, asking for their consent to carry out the research. The WNw-RCT and CTLRC were collectively applied in the classroom in three sessions, one for the WNw-RCT and one for each CTLRC subtest (Reading and LC), allowing for a 1-week interval between tests. Raven’s Test and the RST were individually applied in two sessions of 30 min, one for each instrument. The assessment sessions lasted approximately 30 min each. The assessment occurred in the middle of the school year.

### Statistical Analysis

We performed descriptive and inferential (the ANOVA of the grade effect) statistics for each reading measurement. For groups with significant differences between the performances of the instruments, effect size (ES) analyses were conducted. To investigate the correlations between RC and other measures, we performed a Pearson correlation analysis (with the total sample). A hierarchical linear regression analysis was performed to investigate whether RS has some unique contribution (Model 3) to RC after controlling for intelligence (Model 1), word recognition, and listening (Model 2). To investigate differential correlations between RS and RC as a function of grade level, we performed a Pearson correlation and a partial correlation (controlling for word recognition) analysis independently for each grade level.

## Results

**Table 1** presents descriptive statistics for each measure as a function of grade and for the total sample. Significant effects were found for all measures with increases in scores and speed (and a decrease in response time) as a function of grade. ES analyses found some important effects. For reading speed (RST), ES was moderate between the 2nd, 3rd (*d* = 0.62) and 4th grade (*d* = 0.62). For recognition of words and pseudo words (WNw-RCT), large ESs were found between 2nd and 3rd grade (*d* = 1.15) and between 2nd and 4th grade (*p* = 1.44). For reading comprehension (RC-CTLRC), large ESs were also verified between 2nd and 3rd grade (*d* = 0.97) and 2nd and 4th grade (*p* = 1.12). For listening (CTLRC-LC), large ESs were observed between 2nd and 3rd grade (*d* = 0.86) and between 2nd and 4th (*d* = 0.82).

**Table 1:** Descriptive and inferential statistics of grade effect on listening and reading measurements.

		*N*	Mean	*SD*	*F*(2,211)	*p*	*Post-hoc* analyses
Word recognition	2	85	56.82	5.504	50.780	<0.001	2 < 3, 4
	3	52	62.40	3.560			
	4	75	64.49	5.134			
	Total	212	60.91	6.017			
Reading comprehension	2	85	27.13	11.025	37.153	<0.001	2 < 3, 4
	3	52	35.83	3.730			
	4	75	36.60	3.915			
	Total	212	32.61	8.800			
Listening comprehension	2	85	35.05	4.290	21.826	<0.001	2 < 3, 4
	3	52	38.10	1.660			
	4	75	38.00	2.594			
	Total	212	36.84	3.538			
Reading speed	2	85	0.7824	0.28911	12.579	<0.001	2 > 3, 4
	3	52	0.6324	0.13680			
	4	75	0.6448	0.09567			
	Total	212	0.6969	0.21434			


*Word recognition: score in WNw-RCT – Words and Non-words Reading Competence Test; Reading comprehension: score in CTLRC-RC – Reading Comprehension subtest of the Contrastive Test of Listening and Reading Comprehension; Listening comprehension: score in CTLRC-LC – Listening Comprehension subtest of the Contrastive Test of Listening and Reading Comprehension; Reading speed: one word reading (locution) time (in seconds) in RST – Reading Speed Test.*

**Table 2** presents the correlations between the measurements. RC had a positive significant relationship of a high magnitude with listening; of moderate magnitude with word recognition and a negative significant relationship of low magnitude with RS.

**Table 2:** Correlation matrix between speed, word recognition, listening and reading comprehension (total sample).

		Reading comprehension
Reading speed	*r*	-0.29
	*p*	0.000
Word recognition	*r*	0.58
	*p*	0.000
Listening comprehension	*r*	0.66
	*p*	0.000


Based on the relationships found, we performed a hierarchical regression analysis with RC as a criterion variable. Three models were generated, all with satisfactory fit (*p* < 0.001). **Table 3** shows the models and coefficients for each predictive measure.

**Table 3:** Models from the regression analysis of the prediction of reading comprehension.

Model		Beta	*t*	*p*	*R*^2^	*R*^2^ adjusted
1	(Constant)		16.712	0.000	0.167	0.163
	Intellectual ability	0.408	6.482	0.000		
2	(Constant)		-7.910	0.000	0.533	0.526
	Intellectual ability	0.090	1.661	0.098		
	Word recognition	0.339	6.300	0.000		
	Listening comprehension	0.458	7.887	0.000		
3	(Constant)		-5.776	0.000	0.552	0.544
	Intellectual ability	0.100	1.882	0.061		
	Word recognition	0.305	5.643	0.000		
	Listening comprehension	0.446	7.823	0.000		
	Reading speed	-0.144	-2.989	0.003		


*Intellectual ability: percentile in Raven’s Colored Standard Progressive Matrices.*

Model 1 includes only the intelligence measurement and explains 16% of the variance in RC. Model 2 includes the measurements of listening and word recognition, and the predictive power of the model increased to 52.6%. It is worth noting that with the inclusion of such variables, the contribution of intelligence is no longer significant. Model 3 adds the speed measurement. Although modest, the inclusion of this variable increased the explanatory power of the model in a significant way, and the contribution of RS for RC was significant, despite the control of previous variables, which suggests some unique contribution.

In addition, we explore the relationships between speed reading measurements and RC throughout the grades. As **Table 4** shows, there was a significant, negative, and low relationship between RC and speed only for the 4th grade, showing that children who needed more time in word reading (slower readers) tended to have lower RC. Based on such relationships, we performed a partial correlation analysis between RS and RC, controlling for word recognition. For the 4th grade, the relationship previously found became marginal. **Table 5** shows these results.

**Table 4:** Correlation matrix between reading comprehension and reading speed in each grade level.

School grade	2	*r*	-0.17
		*p*	0.122
	3	*r*	0.12
		*p*	0.409
	4	*r*	**-0.30**
		*p*	**0.009**


*Significant relations highlighted in bold.*

**Table 5:** Partial correlation matrix between reading comprehension and reading speed in each grade level, controlling for word recognition ability.

School grade	2	*r*	-0.14
		*p*	0.198
	3	*r*	0.10
		*p*	0.492
	4	*r*	**-0.21**
		*p*	**0.069**


*Controlling for Word recognition (score in WNw-RCT). Significant relations highlighted in bold.*

## Discussion

The first objective of this study was to investigate the contribution of RS for RC after controlling for intelligence, word recognition, and listening. First, we conducted an ANOVA to verify the development of reading ability during the initial elementary school grades. Results revealed that listening and RC, word recognition, and RS developed during the 2nd, 3rd, and 4th grades, and no significant differences were found between the more advanced grades. These results suggest that, from the 2nd to 3rd grades, there is an important development of reading skills in Brazilian students, and there may be a consolidation in the progression from 3rd to 4th grade. Such differences in performance along grade levels were expected (e.g., Dias et al., 2015) and encouraged conducting some analyses separately for each grade level, as will be discussed later.

The correlation analysis between RC and listening, word recognition, and RS revealed significant relationships in all cases. Considering only reading abilities, as expected, listening and word recognition presented the strongest relationships with RC, while RS was only connected to low magnitude with comprehension. Expanding the results of this analysis, the findings from the regression analysis revealed that even with intelligence controlled, listening and word recognition can explain 52.6% of the variance in RC. This evidence corroborates the value of the components of the SVR (or the cognitive domain of CMR). Indeed, our data virtually replicate the previous findings of Joshi and Aaron (2000), who found that listening and word recognition accounted for 48% of variance in RC in 3rd graders, and Aaron et al. (2008), who found that these abilities explain approximately 40% of RC in students from 2nd to 5th grade.

In addition, Joshi et al. (2012), examining students from 2nd, 3rd, and 4th grades, found that listening and word recognition could explain 60% of the variance in RC for Spanish speakers, while approximately 50% of the variance in RC was explained for English speakers. In our sample, with Portuguese (Brazilian Portuguese) speakers, we found that 52.6% of variance in comprehension can be explained by the component skills. While Spanish has a transparent orthography, English is an opaque orthography. Portuguese has irregularities and rules but in general has a more transparent orthography than English. Despite this, our findings were more similar to results found in English rather than Spanish speakers.

The inclusion of RS in the regression increased the explanatory power of our model to 54.4% (an increase of 1.8%). Although low, the unique contribution of RS to comprehension was significant. Results in this area are debatable. For example, one study found that speed (assessed in a naming speed task) added 10% to the explanatory power of the RC model (Joshi and Aaron, 2000), while other evidence revealed contributions that can vary from 11% in 2nd grade to 2.5% in 5th grade (Aaron et al., 2008) and from 2% for 4th grade and 1.4% for 5th grade (Johnston and Kirby, 2006). Despite having different measures of speed, as used with word RS, our results are very similar to Johnston and Kirby’s (2006), as we also found a low contribution of naming speed (1.8%) for RC models. Studies involving reading fluency (instead of naming speed) also support the relationship between RC and measures of fluency (Klauda and Guthrie, 2008). For instance, Turkyılmaz et al. (2014) found that in 5th grade, fluency (including oral reading fluency, silent reading fluency, and retell fluency) explained 57% of the total variance in RC, but other predictors were not used in this study.

In this sense, our first hypothesis (word RS will present a modest but significant contribution to the explanatory model of RC, showing some unique contribution not accounted for by intelligence, word recognition or listening) proved correct; despite this, we expected the greatest contribution of word RS in our sample with students in the first years of elementary school. It is possible that we were not able to find greater contributions of speed for the model due the fact that speed may already have an effect upon word recognition; or, alternatively, the impact of RS on comprehension should not be relevant, for example, for these younger students, in which the decoding skill is so incipient that there is no variation among students. According to Yovanoff et al. (2005), fluency is a more meaningful measurement when the variation among student reading fluency is maximized. This hypothesis can be elucidated by our second objective, which is to investigate the differential relationships and contributions of RS to comprehension in different school grade levels.

We found a significant relationship between RS and RC only for the 4th grade. Furthermore, when controlling for word recognition, such a relationship was only a marginal trend. No significant relationships were found for 2nd and 3rd grades. The data reveal that RS only begins to be related to reading competence in 4th grade, with no correlation in early stages of elementary school. In this sense, the results corroborated our second hypothesis, which is that faster reading is expected with academic progression.

Regarding this finding, it is interesting to note that Aaron et al. (2008) suggest a trend for the decrease in the effect of speed with academic progression. Additionally, Klauda and Guthrie (2008) hypothesize that word-level fluency could be more connected with comprehension at the beginning of elementary school. Our results indicated the opposite. Klauda and Guthrie (2008) indeed claimed that fluency and comprehension should become more similar (then, more correlated) over time up to the age of 10 or 12, which is the mean age of our 4th grade students. Furthermore, we can use developmental data to explain our results. For example, previous research with Brazilian children suggests that word recognition is better developed only at 4th grade, with more use of orthographic strategy; that is, automaticity (and then speed) may be more important for these students. On the other hand, students from 3rd grade, but mainly 2nd grade, are very dependent on decoding skills, which is a slower process, and at this initial stage of learning, accuracy could be more important than speed to comprehension (Dias et al., 2015). Therefore, the significant relationship in this study between RS and RC only for the 4th grade can be explained because, in these more advanced grades, the importance of alphabetic reading automation and the use of orthographic reading automation increase.

Additionally, according to our results, at least 45% of the variance in RC remains unexplained. In this sense, more studies are needed to clarify other demands of the reading competence model. Our study had some limitations, including the small number of participants, which makes the performance of regression analyses separated by grade level inviable to. Additionally, another limitation concerns the study design, of cohorts of different grade levels, instead a longitudinal study. This design prevents sure whether the observed differences in the relevance of RS for RC are, in fact, due to the child’s developmental stages. Longitudinal studies should be conducted to ensure that the differences observed here are not due to specific characteristics of the study sample. Another limitation concerns the fluency test. We used a word speed test. However, fluency has been defined as a combination between accuracy, automaticity, and prosody (Kuhn et al., 2010); therefore, other tests, such as text RS, can bring different results.

On the other hand, one strength of the current study concerns the language/orthography feature. As most studies on SVR or reading competence models and components are performed with English speaking participants, we expand these data for a different orthography in working with Portuguese speaking students. In conclusion, some educational implications may be extrapolated as the comprehension of cognitive models and theirs components provide some framework when studying and diagnosing reading difficulties, providing professionals with guidelines about what skills to evaluate and how to plan interventions for children with reading difficulties.

## Final Views

Findings suggest that RS can contribute to RC beyond the variance shared with listening and word recognition, but such contributions were low. The data also reveal a differential relationship and contribution of RS in different school grades. Specifically, only in the 4th grade does RS begin to have some association to reading competence. The findings add a developmental perspective to the study of reading models and expand the previous research on reading components and models to a more transparent orthography, such as Portuguese.

## Ethics Statement

The project was approved by the Ethics Committee of the Universidade Sao Francisco, Brazil. Upon approval, the person legally responsible for the child signed the free and informed consent term, according to the rules of the National Council of the Brazil Health. In addition, children expressed verbally their consent to participate. Participation in the study did not offer risks and participants could withdraw at any time.

## Author Contributions

All authors listed, have made substantial, direct and intellectual contribution to the work, and approved it for publication.

## Conflict of Interest Statement

The authors declare that the research was conducted in the absence of any commercial or financial relationships that could be construed as a potential conflict of interest.
